# Ubiquitous Conjugative Mega-Plasmids of *Acinetobacter* Species and Their Role in Horizontal Transfer of Multi-Drug Resistance

**DOI:** 10.3389/fmicb.2021.728644

**Published:** 2021-09-21

**Authors:** Sofia Mindlin, Olga Maslova, Alexey Beletsky, Varvara Nurmukanova, Zhiyong Zong, Andrey Mardanov, Mayya Petrova

**Affiliations:** ^1^Institute of Molecular Genetics of National Research Center “Kurchatov Institute”, Moscow, Russia; ^2^Institute of Bioengineering, Research Center of Biotechnology of the Russian Academy of Sciences, Moscow, Russia; ^3^Center of Infectious Diseases, West China Hospital, Sichuan University, Chengdu, China

**Keywords:** *tra*-operon, replication initiation protein, iterons, plasmid backbone, accessory region, phylogenetic analysis, mobilization

## Abstract

Conjugative mega-plasmids play a special role in adaptation since they carry a huge number of accessory genes, often allowing the host to develop in new niches. In addition, due to conjugation they are able to effectively spread themselves and participate in the transfer of small mobilizable plasmids. In this work, we present a detailed characterization of a recently discovered family of multiple-drug resistance mega-plasmids of *Acinetobacter* species, termed group III-4a. We describe the structure of the plasmid backbone region, identify the *rep* gene and the origin of plasmid replication, and show that plasmids from this group are able not only to move between different *Acinetobacter* species but also to efficiently mobilize small plasmids containing different *mob* genes. Furthermore, we show that the population of natural *Acinetobacter* strains contains a significant number of mega-plasmids and reveal a clear correlation between the living conditions of *Acinetobacter* strains and the structure of their mega-plasmids. In particular, comparison of the plasmids from environmental and clinical strains shows that the genes for resistance to heavy metals were eliminated in the latter, with the simultaneous accumulation of antibiotic resistance genes by incorporation of transposons and integrons carrying these genes. The results demonstrate that this group of mega-plasmids plays a key role in the dissemination of multi-drug resistance among *Acinetobacter* species.

## Introduction

The genus *Acinetobacter* includes species of different life-styles, from free-living saprophytes to human and animal pathogens (Touchon et al., 2014). *Acinetobacter* species occur in diverse natural and artificial environments such as forest and agricultural soils, animal and human skin and gut, fresh- and seawater, or even sewage and activated sludge (Peleg et al., 2012; Touchon et al., 2014). Due to the importance of *Acinetobacter* strains in the clinic, the number of publications devoted to the study of this genus has increased significantly in recent years. The most studied *Acinetobacter* species is the human pathogen *A. baumannii* (Peleg et al., 2012; Salgado-Camargo et al., 2020), which has attracted exceptional attention because of its pathogenicity and multi-drug resistance (Göttig et al., 2014). However, despite their high prevalence in most environments, the distribution and ecological roles of various *Acinetobacter* species, apart from pathogenic and nosocomial species with clinical importance, have remained poorly explored. While non-baumannii acinetobacters live in a wide range of environments including habitats contaminated with heavy metals (Turton et al., 2010; Mindlin et al., 2016), the mechanisms of horizontal gene transfer and, in particular, the role of various groups of plasmids in this process in *Acinetobacter* have not been studied in much detail.

Conjugation is the main process by which genes (including antibiotic−resistance genes) are horizontally transferred from one bacterium to another and is therefore a major contributor to bacterial genome plasticity, evolution and adaptation (Brovedan et al., 2020; von Wintersdorff et al., 2016). Conjugative plasmids play a key role in the physical transfer of DNA from cell to cell. Any conjugative plasmid contains the backbone or core region, a set of genes and elements that ensure its replication, maintenance in the cell and transfer to other cells, and a varying number of accessory genes, which may encode for drug resistance or have other adaptive functions (Thomas, 2000). The number of sequenced *Acinetobacter* plasmids in genomic databases has exceeded 3,000 and continues to grow rapidly. It should be noted that the genus *Acinetobacter* is characterized by the presence of numerous plasmids in the same strain (Feng et al., 2016; Brovedan et al., 2019; Mindlin et al., 2020), and most of them contain the relaxase gene (*mobA*), which suggests their potential ability to be mobilized (Francia et al., 2004; Garcillán-Barcia et al., 2009). Large conjugative plasmids are usually found in the study of clinical antibiotic-resistant strains of *Acinetobacter*, but only some of them are studied in detail. Despite the fact that the ability to carry out conjugative transfer has been confirmed experimentally for several plasmids (Silva et al., 2018; Wibberg et al., 2018), it remained largely unexplored whether they are able to mobilize other non-conjugative plasmids containing the relaxase gene.

To date, three groups of conjugative plasmids are known in *Acinetobacter*, for which their ability to move from one strain to another has been experimentally proven. Each group was formed on the basis of a high level of homology of the backbone regions. A group of plasmids closely related to pACICU2 (64,366 bp) (NC_010606.1) was identified first (Hamidian and Hall, 2014). These plasmids were assigned to the LN_1 lineage in the classification of *A. baumannii* plasmids (Salgado-Camargo et al., 2020). It was shown that pACICU2 contains a complete conjugative apparatus and its relaxase gene belongs to the MOB_*F*_ family. Some plasmids from this group contain the *bla*_*oxa23*_ gene and are widespread mainly in *A. baumannii* strains (Bertini et al., 2010; Nigro et al., 2015). Conjugative plasmids from the second group [prototype pLS488 (NZ_MF078634)] were found in *Acinetobacter* strains belonging to different species, but are less common than representatives of the first group. In most cases they contain antibiotic resistance genes (Silva et al., 2018). All of them contain a complete set of genes involved in the conjugation process and a gene encoding a replication initiator protein. Its relaxase gene belongs to the MOB_*P*_ family. In the work of Mindlin et al. (2020), this group of plasmids was designated III-1a. In contrast, neither the relaxase gene nor the gene encoding the replication initiator protein could be identified in the third group of conjugative mega-plasmids, represented by the prototype plasmid pA297-3 (Hamidian et al., 2016; Nigro and Hall, 2017). At the same time, it was shown that this plasmid is able to actively move between different strains (Hamidian et al., 2016; Nigro and Hall, 2017). The authors believe that the relaxase gene should be present in the plasmid, but the corresponding protein belongs to a new, not yet described relaxase family (Hamidian et al., 2016). It should be noted that other groups of plasmids (for example, related to pAVAci1 or pABTJ1) containing conjugative transport genes are revealed in *Acinetobacter* strains, but the functional activity of these genes remains unknown to date.

At the end of 2020, another group of *Acinetobcater* conjugative plasmids was discovered simultaneously by two teams of researchers (Ghaly et al., 2020; Mindlin et al., 2020). In Mindlin et al. (2020), this group was designated III-4a. It includes mega-plasmids with the size of about 300 kb, which also do not contain known replicase and relaxase genes. It was found that plasmids of this group are widely distributed in predominantly clinical strains of various *Acinetobacter* species. Analysis of the genomes of plasmids from this group suggested that they play an important role in adaptation, since different geographical regions are characterized by their own sets of adaptive genes, while sharing a conserved core genome (Ghaly et al., 2020).

In this work, we performed a detailed characterization of group III-4a plasmids, including: (1) description of the structure of the backbone region; (2) identification of the *rep* gene and the origin of plasmid replication; (3) demonstration that plasmids from group III-4a are conjugative and can efficiently mobilize small plasmids containing different *mobA* genes; (4) data on the wide distribution of plasmids of the III-4a group among environmental strains of *Acinetobacter*.

## Materials and Methods

### Media and Growth Conditions

Bacteria were grown in lysogeny broth (LB) medium or solidified agar LB medium (LA) (Sambrook and Russell, 2001) at 30°C. When required, LB agar was supplemented with antimicrobial agents at the following final concentrations (μg/ml): HgCl_2_ (Hg) 4–5; K_2_Cr_2_O_7_ (Cr) 70–140; streptomycin (Sm) 100–200; chloramphenicol (Cm) 20; gentamycin (Gm) 5; rifampicin (Rif) 25; nalidixic acid (Nal) 20; ceftazidime (Cef) 200; tetracycline (Tc) 10.

### Bacterial Strains and Plasmids

Both mercury resistant (Hg-r) and mercury-sensitive (Hg-s) *Acinetobacter* strains from the IMG collection were used in this study (Supplementary Table 1). The host strain of pALWED1.1 (*A. lwoffii* ED23-35) was isolated from permafrost sample aged forty thousand years. Part of the *Acinetobacter* sp. strains was isolated from mercury mines in different regions of the former Soviet Union. Additional strains from the collections were isolated from soils and water samples from different geographical regions (Supplementary Table 1; Petrova et al., 2002; Kholodii et al., 2004; Mindlin et al., 2005). The strain *A. wuhouensis* WCHAW010062 containing pOXA23_010062 was kindly provided by A. Nemec. The strains *A. baylyi* BD413rif and *A. lwoffii* BSW27-2nal were used as recipients in matings. Small mobilizable plasmids used in this work are presented in Table 1.

**TABLE 1:** Mobilizable plasmids analyzed.

Plasmid	Natural host	MOB family, Group^*^	Resistance to
pALWED 3.5	*A. lwoffii* ED9-5a	MOB_Q_, II-1b	Chromium (Cr)
pALWVS1.4	*A. lwoffii* VS15	MOB_Q_, I-1c	Chloramphenicol (Cm)
p7_010062	*A. wuhouensis* WCHAW010062	MOB_Q_, I-1a	Tetracycline (Tc)
RSF1010	Different gamma-proteobacteria	MOB_Q_, -	Streptomycin (Sm)

*Group number according to plasmid classification in Mindlin et al. (2020).

### Standard DNA Manipulations

Standard protocols were used for agarose gel electrophoresis, and colony hybridization (Sambrook and Russell, 2001). GeneJET Genomic DNA Purification kit (Thermo Fisher Scientific) was used for total genomic DNA isolation. PCR was performed with a Mastercycler (Eppendorf) using Taq DNA polymerase with supplied buffer (Thermo Fisher Scientific) and a dNTP mixture (Thermo Fisher Scientific). The primers trbC-F: ggtctacctgtttatgcatcc and trbC-R: aattcgccgttgtgctgtcc were used to amplify the fragment of *trbC* gene (20950–22249 position in the sequence KX426227) and rep-F: tgtctgaactctctttaccg and rep-R: gtatgcacatcagctgcagc—to amplify the fragment of the putative *rep* gene (228166–229724 position).

### Screening of Plasmids Related to pALWED1.1 Among Modern *Acinetobacter* Strains

We screened 57 environmental strains of *Acinetobacter* isolated in our laboratory from samples of soil and water (Supplementary Table 1). At the first stage of screening, the colonies of all strains were hybridized with a probe (1,300 bp) containing the *trbC* gene encoding the coupling protein (CP) from pALWED1.1 (20950–22249 position in the sequence KX426227). At the second stage of screening, genomic DNA was isolated from all hybridization positive strains and PCR was performed with primers for the putative *rep* gene and the gene *trbC* from pALWED1.1.

### Analysis of the Frequency of Conjugation Transfer of pALWED1.1-Related Plasmids

The ability to transfer resistance markers during conjugation was tested for 6 from 13 strains containing plasmids related to pALWED1.1. These strains were as resistant to mercury as the original strain ED23-35 and one of them was resistant to streptomycin and tetracycline. In addition to them, we tested the conjugative transfer of the plasmid pOXA23_010062 (CP033130.1), also belonging to group III-4a, from the strain *A. wuhouensis* WCHAW010062. All the analyzed strains were crossed with rifampicin-resistant mutants of the *A. baylyi* BD413 strain that does not contain its own plasmids. Matings were performed overnight on the surface of LA plates. Cultures of the donor and recipient in the late logarithmic growth phase were mixed in a ratio of 1: 1; the mixture was plated on the LA surface and incubated at 30° for 18–20 h. The mixed growth was then scrapped off the plate, resuspended, and suitable dilutions were spread on appropriate selective plates. Parent strains were plated in parallel with the matings and then processed similarly to the matings as controls. Isolated colonies from matings and of parental strains were used to identify recombinants and parental forms.

### Mobilization Assays

The small mobilizable plasmids (Table 1) were transformed into *A. baylyi* BD413rif. The conjugative plasmid pALWED1.1 was then transferred to these strains by conjugation with the strain *A. lwoffii* ED23-35. The standard procedure of mating a donor strain harboring two plasmids (conjugative and non-conjugative) with a recipient strain (nalidixic acid-resistant mutant of the strain BSW27-2) was used (Brasch and Meyer, 1986). Matings were performed overnight on the surface of LA plates as described above. Transconjugants were selected on LA plates supplemented with appropriate antimicrobial agents. The mobilization frequency was calculated according to Brasch and Meyer (1986).

### Identification of the Backbone Region of pALWED1.1

The genes involved in conjugation [*mob* genes and mating pair formation (MPF) genes] were identified by amino acid similarity with genes of previously described plasmids. The plasmid R64 (AB027308.1, NC_005014) from *Salmonela enterica* and plasmid pA297-3 (KU744946.1) isolated from *A. baumannii* A297 (Hamidian et al., 2016) were used as references for the MPF I group of the T4SS system and CPT4 from the MOB_*F*_ family.

Previously, neither we nor other researchers (Ghaly et al., 2020) were able to detect the *rep* gene of mega-plasmids. In this paper, we conducted a more careful search. To this end, the backbone regions presented in all mega-plasmids, including an extended region containing the genes involved in conjugation, were determined and hypothetical proteins presented in all plasmids were identified. The identified hypothetical proteins were analyzed using the BLAST Protein on NCBI site (Altschul et al., 1997), which allowed us to find the gene encoding the putative replication initiation protein. The putative iterons of mega-plasmids were revealed manually by the analysis of the region next to the putative *rep* gene.

### Search for Plasmids Related to pALWED1.1 in GenBank

Plasmids related to pALWED1.1 from modern *Acinetobacter* strains were identified using the BLASTp program. The sequence of pALWED1.1 was used as query to search for related plasmids in NCBI database containing complete plasmid genomes on April 1, 2021. All plasmids that had the query cover >50% and the identity of the common region >98.5% were considered as related to pALWED1.1. Since all the detected plasmids contained genes encoding the CP TrbC and the putative replication initiator protein Rep, the sequences of these two genes were used as queries to search for related sequences in the NCBI database containing whole-genome shotgun contigs.

### Bioinformatic Analysis

Phylogenetic trees were built in the following way. First, we constructed multiple alignment of the plasmid complete genomes using Mauve v2.4.0. A Mauve genome alignment results in a set of alignment blocks, each of which is a conserved region across multiple sequences. Alignment blocks present in all plasmid genomes were concatenated and used as an input for tree construction in Phyml v3.3 with default parameters. The concatenated alignment was 352,008 bases in length. Blast comparison between pALWED1.1 and pAHTJR1 plasmid genomes was visualized using Easyfig (Sullivan et al., 2011), alignments with minimum length of 1,000 bp and e value >1e-3 were used.

## Results

### The Molecular Structure of Plasmid pALWED1.1 as a Typical Representative of Group III-4a

Plasmid pALWED1.1 (original designation pKLH208) was isolated from the ancient permafrost strain ED23-35 of *A. lwoffii* resistant to mercury salts (Petrova et al., 2002). It was shown that it is a large plasmid that is able to transfer mercury resistance by conjugation (Kholodii et al., 2004). Initially, only the plasmid region containing the genes of the *mer*-operon encoding mercury resistance was sequenced and studied (Kholodii et al., 2004). Later, thanks to the complete sequencing of pALWED1.1 (287,631 bp) it became possible to study the structure of extended plasmid regions containing determinants of resistance to heavy metal salts (Mindlin et al., 2016). Finally, due to the appearance of a large number of complete genomes of *Àcinetobacter* plasmids, it became clear that pALWED1.1 is a typical representative of an extensive group of plasmids, designated III-4a (Ghaly et al., 2020; Mindlin et al., 2020). However, the structure of the backbone region of the plasmids of this group remained unexplored. Therefore, one of the goals of this work was to describe the structure of the backbone region of plasmids belonging to the group III-4a on the example of pALWED1.1.

### Backbone Region of pALWED1.1

#### Identification of Genes Involved in Conjugation

The transfer of plasmids by conjugation is carried out by several groups of proteins encoded by plasmid genes. The relaxosome complex is responsible for DNA cleavage at the origin of transfer (*oriT*) and formation of relaxosome (Smillie et al., 2010). The MPF complex is involved in the building of pilus and pore necessary for translocation of single-stranded DNA. The MPF complex and relaxosome are linked via the ATPase CP, one of the key proteins of conjugation apparatus (Smillie et al., 2010; Llosa and Alkorta, 2017).

**FIGURE 1:**
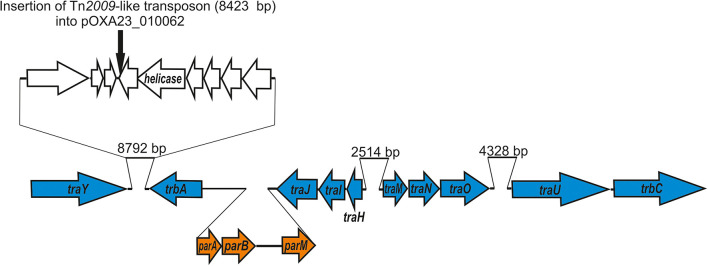
Genetic structure of pALWED1.1 region involved in conjugation. The location and polarity of genes are shown with arrows. Genes of MPF complex are marked in blue; genes encoding partitioning process—in orange. In the top the site of insertion of Tn*2009* (black arrow) into the plasmid pOXA23_010062 is shown.

We identified the putative transfer region of pALWED1.1 and found that it contains a set of MPF genes belonging to the MPF I group of these genes found in other conjugative plasmids (Table 2). In particular, we identified genes *traU* and *traO* as well as other genes necessary for functioning of the T4SS system (Figure 1). All the genes of the MPF module as well as the gene *trbC* encoding the coupling protein T4CP are located in a single plasmid region. Besides the T4SS genes, the genes *parABM* encoding the system of plasmid partitioning are also present in the same region (Figure 1).

**TABLE 2:** Identification of the pALWED1.1 genes involved into the formation of MPF complex.

Gene	Coordinates	Identity (%) of aa sequences found in R64 AB027308.1 (%)	Identity (%) of aa sequences found in pA297-3 (KU744946.1) (%)
*traY*	273149–276157	24	50
*trbA*	284947–286476	–	44
*parA*	296–1132	–	51
*stbA*	3072–4178	–	34
*traJ*	4484–5779	31	49
*traI*	5807–6616	22	37
*traH*	6654–7100	–	39
*traM*	9613–10389	–	38
*traN*	10394–11350	40	43
*traO*	11372–12895	33	38
*traU*	17222–20404	29.7	47
*trbC*	20527–23319	33	40.8

*“–” not found.*

We found homology between the Tra proteins of the pALWED1.1 plasmid and the T4SS system proteins from the R64 plasmid belonging to the MOB_*F*_ family, suggesting that the *tra* genes of this group of mega-plasmids can be placed into the MOB_*F*_ family. However, the gene *mobA* encoding relaxase, the protein necessary for nicking DNA and forming the relaxasome, was not found, and none of the pALWED1.1 plasmid genes showed significant similarities with any of the known relaxase genes. It can therefore be proposed that the relaxase gene of pALWED1.1 belongs to a new not yet described family. Indeed, in some other conjugative plasmids a gene encoding relaxase also has not been identified. Such plasmids in Acinetobacters are the mega-plasmid pA297-3 described by Hamidian et al. (2016) and pNDM-BJ01 and related plasmids described by Hu et al. (2017). Similar observations were made for relaxases of other bacterial plasmids (Smillie et al., 2010; Guzman-Herrador and Llosa, 2019).

#### Identification of the Replication Module of pALWED1.1

**FIGURE 2:**
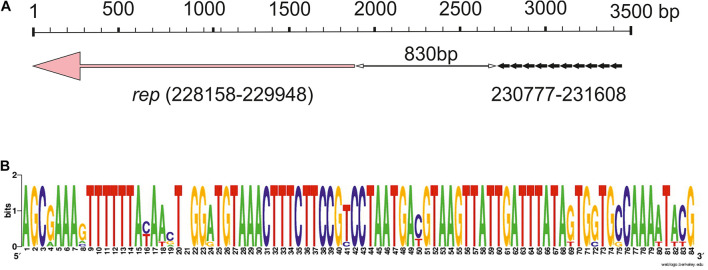
Iteron-controlled putative replicon of pALWED1.1. **(A)** The relative location of the replication initiator gene and the iterons. Pink arrow indicate the *rep* gene, little black arrows indicate surrounding repeat sequences (iterons). **(B)** Consensus sequence of the iterons of pALWED1.1.

In the initial analysis of the pALWED1.1 genome (Mindlin et al., 2016), we were unable to find the gene(s) encoding the protein related to the described plasmid replication initiation proteins. But with a more careful search we found a candidate protein with low similarity to proteins from pfam01051, presumably including plasmid replication proteins, localized in the plasmid region 228158–229948 (Figure 2A). We hypothesize that this particular gene might encode the replication gene. In support of this conclusion, all mega-plasmids from this group contain a gene that is almost identical to the putative *rep* gene of pALWED1.1 in the same region.

Since many plasmid replicons contain directly oriented AT rich sequences near their *rep* genes, iterons, we analyzed the structure of the intergenic region separating the putative *rep* gene of pALWED1.1 from the neighboring genes. It was revealed that a significant part (about 1,200 bp) of this region is rich in adenine and thymine residues (65%). Moreover, at the distance of 830 bp from the start codon of the putative *rep* gene we found 10 tandem repeats, 83–84 bp each (Figure 2A). From the 10 copies revealed, three copies are identical, two differ by 1–2 bp, and the rest by 4–14 bp (Figure 2B). We found such repeats in all mega-plasmids of the III-4a group, and in all cases their number and relative location remain unchanged. Therefore, we assumed that these repeats are plasmid iterons.

It should be noted that most of previously described iterons are 17–22 bp long, and they are located at a close distance (5–200 bp) from the starting codon of the *rep* gene. The number of their copies is usually 4–5 (Bertini et al., 2010), sometimes more (Konieczny et al., 2014). However, significantly longer iterons were found in some plasmids (Page et al., 2001; Gilmour et al., 2004; Konieczny et al., 2014). In particular, a replicon containing twelve 80–81 bp iterons located at a distance of about 500 bp from the *rep* (*repHI2*) gene was discovered in the large conjugative plasmid R478 isolated from *Serratia marcesens* (Gilmour et al., 2004), and cloning experiments suggested their functional activity (Page et al., 2001).

Despite the lack of significant sequence similarity between the plasmids R478 and pALWED1.1, these replicons share many similar features: (i) both contain long iterons (81 and 83 bp, respectively); (ii) the number of iterons in both plasmids significantly exceeds the usual number of short iterons (12 and 10 vs. 3–5); (iii) in both plasmids they are located at a considerable distance from the replicase gene (500 and 830 bp, respectively); (iv) in both plasmids, iterons are located in the region adjacent to the initiation codon of the replicase gene (Figure 2). This suggests that we did probably succeed in identifying the replicon of mega-plasmids of the III-4a group.

#### Backbone Region of Megaplasmids

**FIGURE 3:**
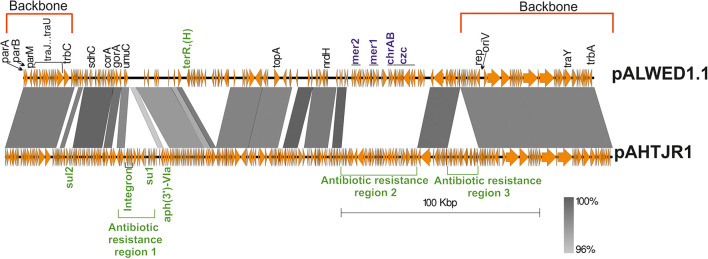
Comparative linear map of plasmids pALWED1.1 and pAHTJR1. The location and polarity of genes and ORFs are shown with arrows. The extent of homologous regions is indicated in the dark gray shading. The backbones regions of plasmids are delimited by square red brackets and antibiotic resistance regions in pAHTJR1—by green. Antibiotic resistance genes are colored in green and genes of resistance to salts of heavy metals—in blue. Antibiotic resistance region 1 contains integron with the cassette genes *arr-3*- (rifamycine resistance) and *aacA4* (aminoglycoside resistance) and the gene *aph(3*″*)-V1a* (aminoglycoside resistance). Antibiotic resistance region 2 contains the genes *oxa58* (carbapenem resistance), the *msrE* and *mphE* (macrolide resistance) and the *floR* (phenicol resistance). Antibiotic resistance region 3 contains the aminoglycosides resistance genes *aph(3*″*)-1b* and *aph(6) -1d* and tetracycline resistance genes *tet*(Y) and *tetR*. Other genes: *sdhC*, succinate dehydrogenase, cytochrome b556 subunit; *corA*, magnesium and cobalt transport protein CorA; *gorA*, glutathione-disulfide reductase; *umuC*, DNA polymerase V subunit UmuC; *topA*, topoisomerase IA; *nrdH*, putative NrdH-redoxin family protein.

The region occupied by the genes of the conjugative complex is highly homologous in all mega-plasmids (Supplementary Table 2). In addition, a significant portion of the region located between the replication initiation control locus and the conjugative complex genes in pALWED1.1 (228.2–287.6 kb) is also present in all related plasmids. Figure 3 shows, using the example of plasmid pAHTJR1, which genes from this region are present in most related plasmids, and which can be replaced. Thus, the main region of this group of mega-plasmids has a total length of 85.2 kb: 1–25 kb (25.8 kb, conjugative genes) and 228.2–287.6 kb (59.4 kb), in the coordinates pALWED1.1 (Figure 3). It is also noteworthy that the non-homologous regions of pALWED1.1 are occupied by heavy metal resistance genes, while those of pAHTJR1 are occupied by antibiotic resistance genes.

### Accessory Regions of Group III-4a Plasmids Carrying Resistance Genes

To obtain more detailed information on the molecular structure and properties of this group of mega-plasmids, we carried out comparative genomic analysis of the 28 plasmids in the size range of 200–300 kb with backbones closely related to those of pALWED1.1 found in the GenBank databases as of April 1, 2021 (Table 3). The main attention was given to the identification of antibiotic and heavy metal resistance determinants and mobile elements that contribute to their spread, and to the study of the conjugative and mobilization properties of these plasmids.

**TABLE 3:** List of pALWED1.1-related mega-plasmids.

Strain	Plasmid	Size, bp	Source	Country/Region	Accession number
*A. lwoffii* ED23-35	pALWED1.1	287,631	Permafrost	Russia: Kolyma	KX426227.1
*A. haemolyticus* TJR01	pAHTJR1	306,131	Human	China: Tianjin	CP038010.1
*A. pittii* 2014N21-145	p2014N21-145-1	323,995	Human	Taiwan	CP033569.1
*A. pittii* C54	pC54_001	256,887	Human	Australia: Sydney	CP042365.1
*A. johnsoni* Acsw19	pAcsw19-2	351,885	Sewage	China: Luzhou	CP043309.1
*Acinetobacter* sp. WCHA55	pOXA58_010055	372,328	Sewage	China: Sichuan, Chengdu	CP032285.1
*A. baumannii* 34AB	p34AB	277,864	Pig (caecum at slaughter)	China: Jiangsu	MK134375.1
*A. pittii* 2014S07-126	p2014S07-126-1	284,051	Human	Taiwan	CP033531.1
*A. wuhouensis* WCHAW010062	pOXA23_010062	311,749	Sewage	China: Sichuan, Chengdu	CP033130.1
*A. defluvii* WCHA30	pOXA58_010030	355,075	Hospital sewage	China: Chengdu, Sichuan	CP029396.2
*A. johnsoni* XBB1	pXBB1-9	398,857	Hospital sewage	China: Chengdu, Sichuan	CP010351.1
*A. baumannii* E47	pE47_001	327,867	Hospital, room 7	Australia: Sydney	CP042557.1
*A. ursingii* RIVM0051	pRIVM0051_IMP-4	259,278	Human	Netherlands: Bilthoven	MH220286
*A. ursingii* RIVM0002	pRIVM0002_IMP-4	317,191	Human	Netherlands: Bilthoven	MH220285
*A. ursingii* RIVM0061	pRIVM0061_IMP-4	313,407	Human	Netherlands: Bilthoven	MH220287
*A. baumannii* ABF9692	pABF9692	264,805	Duck	China: Guangdong province	CP048828.1
*A. pittii* AP43	pAP43-OXA58-NDM1	268,263	Human	China: Hangzhou	CP043053.1
*A. seifertii* AS4	pAS4-1	276,086	Human	Taiwan	CP061688.1
*A. seifertii* AS23	pAS23-1	290,682	Human	Taiwan	CP061673.1
*A. seifertii* AS70	pAS70-1	281,459	Human	Taiwan	CP061572.1
*A. seifertii* AS74	pAS74-1	336,046	Human	Taiwan	CP061557.1
*A. nosocomialis*	pWM08B	255,232	Human	Australia	MT742183
*A. lwoffii*	pR4WN_12CE1	270,906	Prawn	East Australian Fisheries	MT742180
*Acinetobacter* sp. TTH0-4	pR4WN_IBD1	284,751	Prawn	East Australian Fisheries	MT742182
*A. johnsoni*	pR4WN_E10B	259,080	Prawn	East Australian Fisheries	MT742181
*A. pittii* JXA13	pHNJXA13-1	206,931	Dog	China; Nanchang	CP054138
*A. baumannii* ABF9692	pAB9692	264,805	Trachea of duck	China	CP048828.1
*Acinetobacter* sp. CS-2	unnamed2	283,930	Hospital wastewater	China	CP67021.1

Bioinformatic analysis conducted by Ghaly et al. (2020) showed that different variants of the group III-4a plasmids from strains living within the same geographical region usually have a similar structure of accessory regions, whereas in their core genomes they are often evolutionarily distant members of group III-4a. In addition, the authors provided a brief characterization of the accessory regions of group III-4a plasmids, including the total number and distribution of antibiotic resistance genes and associated mobile genetic elements (integrons and MITEs, miniature inverted-repeat transposable elements), as well as heavy metal resistance genes (Ghaly et al., 2020). In this paper, we focus on some features of the genetic structure of the accessory regions of plasmids of this group not described previously.

**FIGURE 4:**
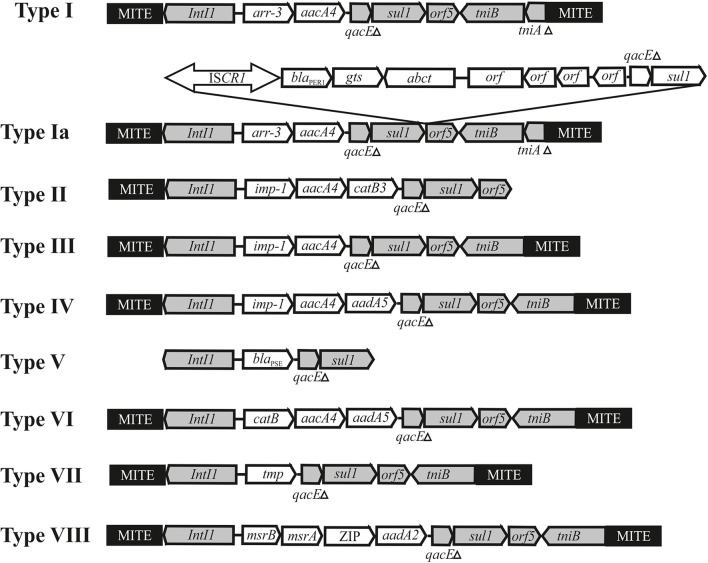
Genetic structure of integrons found in Acinetobacter mega-plasmids belonging to the group III-4a. The location and polarity of genes are shown with arrows. The conserved regions of integrons: 5′-conserved segment—intI1-integrase and 3′-conserved segment—*qacEΔ*, *sul1*, *orf5*, *tniB*, *tniAΔ*. Cassette genes: *arr3*—rifamycin-resistance; *aacA4*, *aadA2* and *aadA5*—aminoglycoside resistance; *catB*, *catB3*—phenicols resistance*; bla*_*PSE*_—beta-lactam resistance; *imp-1*—metallo-β-lactamase, *msrA* and *msrB*—peptide methionine sulfoxide reductase, ZIP-cation transport. MITE–a miniature inverted-repeat transposable elements facilitating mobilization of integron by transposition. Other elements: IS*CR1*-*bla*_*PER*__–__1_ contains gene *bla*_*PER*__–__1_ encoding beta-lactamase.

It was revealed that while having similar backbone regions, the plasmids differ significantly in the structure of accessory regions. Thus, some plasmids (7 out of 28) retain the mercury resistance operon(s) in their genome (Supplementary Table 3) that are part of remnants of transposons that are unable to transpose (Kholodii et al., 2004). At the same time, all plasmids contain genes for resistance to various antibiotics, the set of which differs in various plasmids. It was shown that most clinical plasmids contain a kanamycin resistance transposon (Tn*aph6*), two plasmids (pOXA23_010062 and pAS74-1) contain the transposon Tn*2009* with the *bla*_*OXA*__–__23_ gene, 14 from 20 plasmids don’t carrying mercury resistance determinants contain classI integrons with various set of antibiotic resistance genes (Supplementary Table 3 and Figure 4). It should be noted that most of the integrons are flanked by 439 bp MITEs likely facilitating mobilization of the integron by transposition (Gillings et al., 2009; Domingues et al., 2011, 2013).

The most complex mosaic structure was revealed in the plasmid pXBB1-9 (Zong, 2014), containing a complex Tn*402*-like class 1 integron (Ia) with the *arr3* and *aacA4* cassettes. In addition, it contains a 5.7 kb fragment with IS*CR1* and the metallo-beta-lactamase (*bla*_*PER1*_) gene. The same genetic element (type Ia) is found in the plasmids pAHTJR1 and pOXA58_010055 (Supplementary Table 3). The mechanism of the acquisition of the IS*CR1*-*bla*_*PER*__–__1_ region is not completely clear (Zong, 2014).

The rest of the integrons present in plasmids of group III-4a have a standard structure, except that they contain MITE elements on the flanks. The integrons differ between themselves in the number and set of gene cassettes (Supplementary Table 3 and Figure 4). Sometimes one of the MITE copies is absent (for instance, see integron type II in the plasmid pC54_001 from A. *pittii* C54, Figure 4). In most cases, plasmids contain a single integron. The exceptions are plasmids found in *A. ursingii* strains which contain two or three different integrons. For example, three integrons of plasmid pRIVM0061_IMP-4 from strain RIVM0061 of *A. ursingii* contain cassette genes *arr-3*-*aacA4*, *IMP-4*-*aacA4*-*catB3*, and *bla*_*PSE*_, correspondingly (Supplementary Table 3). In this case, the first of the integrons (type I) is located at a considerable distance from the other two (types II and V), located next to each other. Noteworthy, both MITE copies in second and third integrons are absent. It should be also noted that two from three integrons contain distinct genes for beta lactam resistance (*bla*_*IMP*__–__4_; *bla*_*PSE*_), encoding functionally different proteins.

It is interesting to note that the type VIII intregron, which was only found in plasmids pR4WN_12CE1, pR4WN_IBD1 and pR4WN_E10B from prawns, contain four cassette genes and three of them unrelated to antibiotic resistance and possibly involved in the cell metabolism. These were *msrA* and *msrB*, encoding peptide methionine sulfoxide reductase, the gene encoding an organic cation transport protein that mediates the transport of organic cations across the cell membrane, and *aadA* responsible to streptomycin/spectinomycin resistance encoding aminoglycoside 3″-adenyl-transferase. Interestingly, while the *msrA* and *msrB* genes are commonly found in plasmids or in chromosomes (Koepsell et al., 2003), we could not find the gene encoding the organic cations transport protein in *Acinetobacter* strains, apart from this integron found in strains inhabiting prawns. It can be assumed that the transport protein contributes to the survival of the corresponding *Acinetobacter* strains in prawns.

While nine plasmids related to plasmid pALWED1.1 did not contain integrons, they were also characterized by multiple resistance to antibiotics, due to the presence of transposons and various determinants of resistance. At the same time, most plasmids of this group lacked the determinants of resistance to mercury or other heavy metals, and some contained incomplete sets of the metal resistance genes.

Due to the diversity of the mega-plasmid habitat, we tried to determine the presence / absence of a relationship between the habitat conditions of plasmids and the structure of their genome. For this purpose, two groups of mega-plasmids were selected and compared: (1) plasmids originating from clinical strains of *Acinetobacter* isolated from humans (13 strains) and (2) plasmids originating from environmental strains from sewages and permafrost (7 strains). Although this division into groups is quite conditional, clear differences between strains of the two groups were revealed. Most of the strains of the first group (10 out of 13) contained integrons and only three of them were characterized by resistance to mercury. In contrast, the majority of plasmids of the second group (5 from 7) did not contain integrons and the most of them were resistant to mercury (5 from 7). It should be noted that all modern plasmids were characterized by multiple resistance to antibiotics unlike a permafrost plasmid. Nevertheless, plasmids from wastewater on average contained 1–2 less resistance genes than those isolated from humans. Thus, it can be assumed that the process of adaptation of environmental *Acinetobacter* strains to the existence in the clinic was accompanied by the loss of resistance to mercury and the acquisition of integrons and of multiple resistance to antibiotics. This was achieved by inserting various mobile elements (transposons, integrons) into the plasmid genome. Interestingly, some plasmids contain two and even three integrons.

### Distribution of Group III-4a Plasmids Among Modern Strains

We analyzed the distribution of plasmids from the group III-4a among modern strains of *Acinetobacter*. It was previously shown that these plasmids are widely found in the sequenced genomes of clinical strains of *Acinetobacter* (Ghaly et al., 2020; Mindlin et al., 2020). It should be noted that the number of complete genomes of plasmids of group III-4a is growing rapidly: in addition to 21 mega-plasmids present in the fall of 2020 (Ghaly et al., 2020), 7 more sequences were added until April 1, 2021, thus bringing their total number to 28 (Table 3). In addition, we found the *trbC* and putative *rep* genes, belonging to the backbone region of the mega-plasmids of this group, in unassembled genomes of 59 *Acinetobacter* strains deposited in the GenBank (Supplementary Table 4). Since most *Acinetobacter* strains in the database are of clinical origin, we also screened our collection of *Acinetobacter* environmental strains for the group III-4a plasmids (section “Materials and Methods”). Of the 56 tested strains 14 contained simultaneously the *trbC* and putative *rep* genes highly similar to pALWED1.1 (Supplementary Table 1). Thus, plasmids from group III-4a are widely distributed among both clinical and environmental strains of *Acinetobacter*.

### Functional Activity of Plasmids From Group III-4a

We previously showed that the plasmid pAWED1.1 not only moves itself with a frequency of 8×10^–3^ from the original strain of *A. lwoffii* ED23-35 to the cells of *A. baylyi* BD413rif, but also mobilizes the small plasmid pALWED1.8 (MOB_*HEN*_ family, group I-2b) contained in the same strain, with a similar frequency of 3×10^–3^ (Kurakov et al., 2016). In this work, we investigated the ability of pAWED1.1 to mobilize *Acinetobacter* plasmids belonging to different groups of the MOB_*Q*_ family (Table 1), according to the classification of mobilizable plasmids developed by us (Mindlin et al., 2020), and also checked the conjugation activity of other plasmids from group III-4a.

It was found that pALWED1.1 was able to mobilize all the small *Acinetobacter* plasmids studied, although mobilization events were less efficient than conjugative transfer, which is consistent with the observations of Brasch and Meyer (1986). The frequency of mobilization was different (Table 4). The transfer of the pALWVS1.4 plasmid occurred with a frequency, which was 20 times lower than that of pALWED1.1 itself, while the frequency of transfer of the p7_010062 plasmid was 100 times lower. We also tested the possibility of mobilizing a wide-host range plasmid RSF1010, whose derivatives are widely distributed in clinical strains of various gamma-proteobacteria. Plasmid pALWED1.1 did not mobilize RSF1010. Hence, the pALWED1.1 conjugation system is able to mobilize only *Acinetobacter* plasmids that belong to different groups of the MOB_*Q*_ and MOB_*HEN*_ families.

**TABLE 4:** Mobilization of different small plasmids by pALWED1.1.

Mating		Transconjugants frequency		Ratio
		(per recipient)^*^		
Donor	Recipient	pALWED1.1 (A)	Small plasmids (B)	A/B
BD413rif (p7_010062 + pALWED1.1)	BSW27-2nal	2.6 × 10^–4^	2.3 × 10^–6^	116
BD413rif (pALWVS1.4 + pALWED1.1)	BSW27-2nal	6.8 × 10^–4^	2.9 × 10^–5^	23
BD413rif (pALWED 3.5 + pALWED1.1)	BSW27-2nal	3.0 × 10^–3^	4.0 × 10^–5^	75
BD413rif (RSF1010 + pALWED1.1)	BSW27-2nal	6.0 × 10^–4^	<1 × 10^–8^	–

*Average of three experiments.

The backbone regions of all mega-plasmids belonging to group III-4a includes genes of the conjugative complex (Supplementary Table 2). Unfortunately, the ability of these plasmids to conjugate has not been previously investigated. Since, in addition to pALWED1.1, we had at our disposal another related mega-plasmid with a known nucleotide sequence, pOXA23_010062, we also determined its conjugation transfer frequency. The conjugation transfer of the pOXA23_010062 occurred at a frequency of 2.0×10^–7^, which is four orders of magnitude less than that of pALWED1.1. The reason for this was established by a detailed comparison of the structure of the genomes of the two plasmids. It turned out that the pOXA23_010062 genome, in contrast to pALWED1.1, contained an insertion of the Tn*2009*-like transposon carrying the bla_*OXA23*_ gene (Figure 1). It is essential that the insertion occurred into a gene located next to the *traY* gene, i.e., in the region where the main genes of the conjugative complex are located. At the same time, the available data on the widespread distribution of closely related mega-plasmids leave no doubt that most of them are highly conjugative.

To confirm this assumption, we tested the ability of seven strains in which we found genes *trbC* and *rep* similar to pALWED1.1 (see above) to transmit their resistance markers due to conjugation. It turned out that all strains are able to transmit markers of resistance to mercury or antibiotics to the *A. baylyi* BD413 (Table 5). In most strains, the transfer frequency was similar to pALWED1.1, and in two, NC13-1 and LS12-1, it was drastically reduced. Perhaps this is related with the presence of changes in the structure of their *tra* operons, similar to what we found in pOXA23_010062. All the data obtained indicate that the majority of plasmids from group III-4a are active disseminators of genetic information between cells of different strains of *Acinetobacter* both in the clinical and environmental settings.

**TABLE 5:** Frequency of conjugative transfer of environmental plasmids from the group III-4a.

Mating		Transconjugants frequency (per recipient)^*^
Donor	Recipient	
ED45-25	BD413rif	4.5 × 10^–3^
KHP18	BD413rif	2.5 × 10^–3^
NC13-1	BD413rif	1.5 × 10^–6^
LS12-1	BD413rif	2.3 × 10^–7^
Z13-16	BD413rif	2.2 × 10^–1^
W14	BD413rif	5.5 × 10^–2^
ANS7-7 (Tc-R)	BD413rif	2.7 × 10^–3^
ANS7-7 (Str-R)	BD413rif	6.2 × 10^–2^
WCHAW010062	BD413rif	2.0 × 10^–7^

*Average of three experiments. To rule out that the transconjugants were not spontaneous rifampicin mutants of donor cells the morphological characters of isolated colonies from matings and donor cells were compared because these in parental strains are differed significantly.

### Phylogeny and Evolution of the Plasmids Belonging to the Group III-4a

**FIGURE 5:**
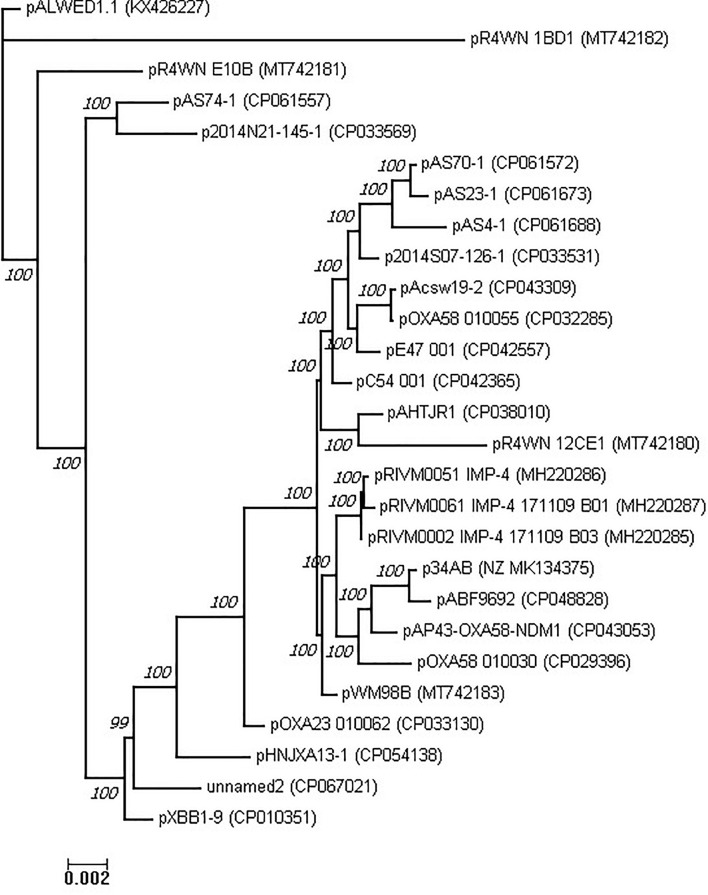
Phylogenetic tree of *Acinetobacter* mega-plasmids (see section “Materials and Methods”).

Previously, Ghaly et al. (2020) used a total of eight genes belonging to the plasmid core genome for phylogenetic analysis of the group III-4a plasmids. In our analysis, we constructed multiple alignment of the complete genomes of 28 plasmids of this group. The concatenated alignment was 352,008 bases in length. This alignment was used to construct a phylogenetic tree of the group III-4a plasmids (Figure 5). In general, the topology of the tree obtained by us coincides with the topology of the tree obtained in the previous work on eight genes.

Apparently, most modern plasmids found in clinical and veterinary isolates share a common ancestor. Nevertheless, judging by the topology of the tree, there are several variants of such plasmids, some of which are found in the clinic and some in nature. It is noteworthy that the environmental variants of the representatives of group III-4a are quite remote from all clinical variants, and the most remote of them is pALWED1.1, isolated from permafrost aged forty thousand years.

## Discussion

The role of plasmids as the main genetic elements involved in the process of horizontal gene transfer has been repeatedly demonstrated by various researchers (Stokes and Gillings, 2011; Martins et al., 2015; Da Silva and Domingues, 2016; Pagano et al., 2016). This is especially evident in acinetobacters, which are characterized by the presence of numerous plasmids in one strain (Feng et al., 2016; Brovedan et al., 2019; Veress et al., 2020). Recently, a novel family of mega-plasmids, designated group III-4a, was discovered, which are ubiquitous among various strains and species of the *Acinetobacter* genus (Ghaly et al., 2020; Mindlin et al., 2020). It turned out that these mega-plasmids are characterized by multiple drug resistance due to the presence of various transposons and integrons in their genomes, along with individual resistance genes. Various combinations of resistance determinants were observed in different members of the group, with a significant diversity in the composition of accessory regions in different plasmids (Ghaly et al., 2020). The role of these plasmids in the propagation of resistance genes among various species of *Acinetobacter* genus was also demonstrated (Ghaly et al., 2020).

In this work, we have filled important gaps left by the previous studies. In particular, a previously unknown replication initiator *rep* gene was in the region 228158–229948 bp of pALWED1.1. The identity of the *rep* gene is confirmed by: (1) the presence of almost identical genes in all mega-plasmids (2) the presence of iterons in the vicinity of the putative replicase gene; (3) the relationship of the putative Rep to the proteins members of the pfam01051, presumably including replicases. Obviously, further research is needed to definitively prove that the selected gene encodes a replication initiator protein.

Despite the fact that we were able to identify a number of genes of the conjugative complex, a relaxase gene related to the known ones was not found in mega-plasmids from the III-4a group. Since other plasmids are known, in which relaxases have not been found (Smillie et al., 2010; Hu et al., 2017; Hamidian et al., 2016), it can be assumed that a larger diversity of relaxases exists in nature that remain to be identified in future studies.

We performed comparative analysis of the structure of the accessory region in 28 sequenced mega-plasmids of III-4a group (Supplementary Table 3). Some of these were isolated from clinical specimens, others from waste water, and the rest from various animal and environmental sources. The main differences between modern plasmids from the III-4a group from the ancient plasmid pALWED1.1 are (1) the complete or partial absence of determinants of resistance to heavy metal salts, and (2) the presence of multiple determinants of resistance to antibiotics. At the same time, the backbone regions of these plasmids are highly homologous.

Analysis of *Acinetobacter* whole-genome shotgun contigs of clinical strains deposited in the Genbank showed that plasmids belonging to the group III-4a are present in 59 genomes. In the collection of environmental *Acinetobacter* strains, group III-4a plasmids are also found in more than 20% of the strains, including ancient isolates from permafrost. The discovery of this group of plasmids in the permafrost samples indicates their wide distribution long before the use of antibiotics. Most likely, similarly to pALWED1.1, ancient plasmids of this group contained various determinants of resistance to heavy metals, since such genes predominate in the composition of large plasmids of five *A. lwoffii* strains isolated from permafrost (Mindlin et al., 2016). After the beginning of the use of antibiotics, the selection of plasmid variants that already contained drug resistance genes or acquired them by horizontal gene transfer, with simultaneous loss of metal resistance genes, has begun. Subsequently, they repeatedly and independently acquired different versions of integrons, which facilitated the process of adaptation to the clinical conditions of the host strains.

## Conclusion

In this work we studied the structure of basic region of multiply resistant mega-plasmids of acinetobacters belonging to the recently discovered group (lineage) named III-4a. A previously unknown gene encoding a replication initiator protein was identified, with 10 copies of 82–83 bp iterons next to it. A number of genes involved in the process of plasmid conjugation and belonging to the MOB_*F*_ family were also identified. The ability of mega-plasmids both to conjugate and to mobilize small *Acinetobacter* plasmids was demonstrated in mating experiments.

Our analysis showed that all sequenced mega-plasmids of this group have a common region of about 85 kb, which includes not only the genes responsible for replication, maintenance and conjugation transfer, but also additional genes not identified until now. It was shown that accessory regions of plasmids contain adaptive genes, including genes for antibiotic resistance, the set of which varies depending on the conditions of existence of the host strain.

Phylogenetic analysis revealed that all clinical and some modern environmental plasmids form one large branch, while most environmental plasmids, including ancient ones, are much more diverse. Our data clearly indicate that conjugative plasmids from group III-4a are widely distributed on all continents, including Antarctica, not only in clinical but also in natural habitats. Such plasmids were also widely and universally distributed tens of thousands years ago. These data, in addition to those obtained earlier (Petrova et al., 2014; Mindlin et al., 2020) add new evidence of the origin of mobile elements of modern clinical bacteria from those of environmental bacteria.

## Data Availability Statement

The datasets presented in this study can be found in online repositories. The names of the repository/repositories and accession number(s) can be found in the article/Supplementary Material.

## Author Contributions

SM had the initial idea, which was developed into a project together with MP. ZZ isolated the strain *A. wuhouensis* WCHAW010062 and SM, MP isolated the other strains used in the research. AM, AB, ZZ, and MP conducted the sequencing, assembly of plasmids, and genome annotation. OM conducted a plasmid screening among a collection of environmental *Acinetobacter* strains. OM and VN conducted the experiments on conjugation and mobilization. AM, AB, and MP performed the bioinformatic analysis. SM, OM, and MP designed the tables. AB and MP processed the figures. SM, MP, and AM wrote the manuscript. All authors approved the submitted version.

## Conflict of Interest

The authors declare that the research was conducted in the absence of any commercial or financial relationships that could be construed as a potential conflict of interest.

## Publisher’s Note

All claims expressed in this article are solely those of the authors and do not necessarily represent those of their affiliated organizations, or those of the publisher, the editors and the reviewers. Any product that may be evaluated in this article, or claim that may be made by its manufacturer, is not guaranteed or endorsed by the publisher.

## Supplementary Material

Supplementary Table 1 Screening of pALWED1.1 plasmid among environmental *Acinetobacter* strains.

Supplementary Table 2 Identification of backbone region genes in genomes of modern plasmids related to pALWED1.1 (summary information for 27 modern plasmids).

Supplementary Table 3 Characteristics of *Acinetobacter* conjugative mega-plasmids.

Supplementary Table 4 *rep* and *trbC* genes of pALWED1.1 in whole genome shotgun sequences.

## References

[B1] Altschul S. F. Madden T. L. Schäffer A. A. Zhang J. Zhang Z. Miller W. (1997). Gapped BLAST and PSI-BLAST: a new generation of protein database search programs. *Nucleic Acids Res.* 25 3389–3402. 10.1093/nar/25.17.3389 9254694 PMC146917

[B2] Bertini A. Poirel L. Mugnier P. D. Villa L. Nordmann P. Carattoli A. (2010). Characterization and PCR-based replicon typing of resistance plasmids in *Acinetobacter baumannii*. *Antimicrob. Agents Chemother.* 54 4168–4177. 10.1128/AAC.00542-10 20660691 PMC2944597

[B3] Brasch M. A. Meyer R. J. (1986). Genetic organization of plasmid R1162 DNA involved in conjugative mobilization. *J. Bacteriol.* 167 703–710. 10.1128/jb.167.2.703-710.1986 3525520 PMC212946

[B4] Brovedan M. Repizo G. D. Marchiaro P. Viale A. M. Limansky A. (2019). Characterization of the diverse plasmid pool harbored by the blaNDM-1- containing *Acinetobacter bereziniae* HPC229 clinical strain. *PLoS One* 14:e0220584. 10.1371/journal.pone.0220584 31743332 PMC6863613

[B5] Brovedan M. A. Cameranesi M. M. Limansky A. S. Morán-Barrio J. Marchiaro P. Repizo G. D. (2020). What do we know about plasmids carried by members of the *Acinetobacter* genus? *World J. Microbiol. Biotechnol.* 36:109. 10.1007/s11274-020-02890-7 32656745

[B6] Da Silva G. J. Domingues S. (2016). Insights on the horizontal gene transfer of carbapenemase determinants in the opportunistic pathogen *Acinetobacter baumannii*. *Microorganisms.* 4:29. 10.3390/microorganisms4030029 27681923 PMC5039589

[B7] Domingues S. Nielsen K. M. da Silva G. J. (2011). The blaIMP-5-carrying integron in a clinical *Acinetobacter baumannii* strain is flanked by miniature inverted-repeat transposable elements (MITEs). *J. Antimicrob. Chemother.* 66 2667–2668. 10.1093/jac/dkr327 21810834

[B8] Domingues S. Toleman M. A. Nielsen K. M. da Silva G. J. (2013). Identical miniature inverted repeat transposable elements flank class 1 integrons in clinical isolates of *Acinetobacter* spp. *J. Clin. Microbiol.* 51 2382–2384. 10.1128/JCM.00692-13 23596242 PMC3697652

[B9] Feng Y. Yang P. Wang X. Zong Z. (2016). Characterization of *Acinetobacter johnsonii* isolate XBB1 carrying nine plasmids and encoding NDM-1, OXA-58 and PER-1 by genome sequencing. *J. Antimicrob. Chemother.* 71 71–75. 10.1093/jac/dkv324 26462992

[B10] Francia M. V. Varsaki A. Garcillán-Barcia M. P. Latorre A. Drainas C. la Cruz F. (2004). A classification scheme for mobilization regions of bacterial plasmids. *FEMS Microbiol. Rev.* 28 79–100. 10.1016/j.femsre.2003.09.001 14975531

[B11] Garcillán-Barcia M. P. Francia M. V. de la Cruz F. (2009). The diversity of conjugative relaxases and its application in plasmid classification. *FEMS Microbiol. Rev.* 33 657–687. 10.1111/j.1574-6976.2009.00168.x 19396961

[B12] Ghaly T. M. Paulsen I. T. Sajjad A. Tetu S. G. Gillings M. R. (2020). A novel family of *Acinetobacter* mega-plasmids are disseminating multi-drug resistance across the globe while acquiring location-specific accessory genes. *Front Microbiol.* 11:605952. 10.3389/fmicb.2020.605952 33343549 PMC7738440

[B13] Gillings M. R. Labbate M. Sajjad A. Giguère N. J. Holley M. P. Stokes H. W. (2009). Mobilization of a Tn402-like class 1 integron with a novel cassette array via flanking miniature inverted-repeat transposable element-like structures. *Appl. Environ. Microbiol.* 75 6002–6004. 10.1128/AEM.01033-09 19648375 PMC2747878

[B14] Gilmour M. W. Thomson N. R. Sanders M. Parkhill J. Diane E. Taylor D. E. (2004). The complete nucleotide sequence of the resistance plasmid R478: defining the backbone components of incompatibility group H conjugative plasmids through comparative genomics. *Plasmid* 52 182–202. 10.1016/j.plasmid.2004.06.006 15518875

[B15] Göttig S. Gruber T. M. Higgins P. G. Wachsmuth M. Seifert H. Kempf V. A. (2014). Detection of pan drug- resistant *Acinetobacter baumannii* in Germany. *J Antimicrob. Chemother.* 69 2578–2579. 10.1093/jac/dku170 24833751

[B16] Guzman-Herrador D. L. Llosa M. (2019). The secret life of conjugative relaxase. *Plasmid* 104:102415. 10.1016/j.plasmid.102415 31103521

[B17] Hamidian M. Ambrose S. J. Hall R. M. (2016). A large conjugative *Acinetobacter baumannii* plasmid carrying the sul2 sulphonamide and strAB streptomycin resistance genes. *Plasmid.* 8 43–50. 10.1016/j.plasmid.2016.09.001 27601280

[B18] Hamidian M. Hall R. M. (2014). pACICU2 is a conjugative plasmid of *Acinetobacter* carrying the aminoglycoside resistance transposon TnaphA6. *J. Antimicrob. Chemother.* 69 1146–1148. 10.1093/jac/dkt488 24335352

[B19] Hu Y. Feng Y. Zhang X. Zong Z. (2017). *Acinetobacter defluvii* sp. nov., recovered from hospital sewage. *Int. J. Syst. Evol. Microbiol.* 67 1709–1713. 10.1099/ijsem.0.001847 28211316

[B20] Kholodii G. Mindlin S. Gorlenko Z. Petrova M. Hobman J. Nikiforov V. (2004). Translocation of transposition-deficient (TndPKLH2-like) transposons in the natural environment: mechanistic insights from the study of adjacent DNA sequences. *Microbiology* 150 (Pt 4) 979–992. 10.1099/mic.0.26844-0 15073307

[B21] Koepsell H. Schmitt B. M. Gorboulev V. (2003). Organic cation transporters. *Rev. Physiol. Biochem. Pharmacol.* 150 36–90. 10.1007/s10254-003-0017-x 12827517

[B22] Konieczny I. Bury K. Wawrzycka A. Wegrzyn K. (2014). Iteron Plasmids. *Microbiol. Spectr.* 2 10.1128/microbiolspec.PLAS-0026-2014 26104462

[B23] Kurakov A. Mindlin S. Beletsky A. Shcherbatova N. Rakitin A. Ermakova A. (2016). The ancient small mobilizable plasmid pALWED1.8 harboring a new variant of the non-cassette streptomycin/spectinomycin resistance gene aadA27. *Plasmid* 84-85 36–43. 10.1016/j.plasmid.2016.02.005 26896789

[B24] Llosa M. Alkorta I. (2017). Coupling proteins in type IV secretion. *Curr. Top. Microbiol. Immunol.* 413 143–168. 10.1007/978-3-319-75241-9_6 29536358

[B25] Martins N. Picão R. C. Adams-Sapper S. Riley L. W. Moreira B. M. (2015). Association of class 1 and 2 integrons with multidrug-resistant *Acinetobacter baumannii* international clones and *Acinetobacter nosocomialis* isolates. *Antimicrob. Agents Chemother.* 59 698–701. 10.1128/AAC.02415-14 25348522 PMC4291415

[B26] Mindlin S. Beletsky A. Rakitin A. Mardanov A. Petrova M. (2020). *Acinetobacter* plasmids: diversity and development of classifications strategies. *Front. Microbiol.* 11:588410. 10.3389/fmicb.2020.588410 33304332 PMC7693717

[B27] Mindlin S. Minakhin L. Petrova M. Minakhina S. Gorlenko Zh Nikiforov V. (2005). Present-day mercury resistance transposons are common in bacteria preserved in permafrost grounds since the Upper Pleistocene. *Res. Microbiol.* 156 994–1004. 10.1016/j.resmic.2005.05.011 16084067

[B28] Mindlin S. Petrenko A. Kurakov A. Beletsky A. Mardanov A. Petrova M. (2016). Resistance of ancient and modern *Acinetobacter lwoffii* strains to heavy metals and arsenic revealed by genome analysis. *Bio. Med. Res. Int.* 3970831. 10.1155/2016/3970831 27795957 PMC5067307

[B29] Nigro S. J. Hall R. M. (2017). A large plasmid, pD46-4, carrying a complex resistance region in an extensively antibiotic-resistant ST25 *Acinetobacter baumannii*. *J Antimicrob Chemother.* 72 3496–3498. 10.1093/jac/dkx287 28961764

[B30] Nigro S. J. Holt K. E. Pickard D. Hall R. M. (2015). Carbapenem and amikacin resistance on a large conjugative *Acinetobacter baumannii* plasmid. *J. Antimicrob. Chemother.* 70 1259–1261. 10.1093/jac/dku486 25433005 PMC4356202

[B31] Pagano M. Martins A. Barth A. (2016). Mobile genetic elements related to carbapenem resistance in *Acinetobacter baumannii*. *Braz J Microbiol.* 47 785–792. 10.1016/j.bjm.2016.06.005 27522927 PMC5052331

[B32] Page D. T. Whelan K. F. Colleran E. (2001). Characterization of two autoreplicative regions of the IncHI2 plasmid R478: rephi2a and RepHI1A (R478). *Microbiology* 147 1591–1598. 10.1099/00221287-147-6-1591 11390690

[B33] Peleg A. Y. de Breij A. Adams M. D. Cerqueira G. M. Mocali S. Galardini M. (2012). The success of *Acinetobacter* species; genetic, metabolic and virulence attributes. *PLoS One* 7:e46984. 10.1371/journal.pone.0046984 23144699 PMC3483291

[B34] Petrova M. Kurakov A. Shcherbatova N. Mindlin S. (2014). Genetic structure and biological properties of the first ancient multiresistance plasmid pKLH80 isolated from a permafrost bacteria. *Microbiology* 160 2253–2263. 10.1099/mic.0.079335-0 25063046

[B35] Petrova M. A. Mindlin S. Z. Gorlenko Z. M. Kaliaeva E. S. Soina V. S. Bogdanova E. S. (2002). Mercury-resistant bacteria from permafrost sediments and prospects for their use in comparative studies of mercury resistance determinants. *Genetika* 38 1569–1574. 12500685

[B36] Salgado-Camargo A. D. Castro-Jaimes S. Rosa-Maria Gutierrez-Rios R.-M. Lozano L. F. Altamirano-Pacheco L. Silva-Sanchez J. (2020). Structure and evolution of *Acinetobacter baumannii* plasmids. *Front. Microbiol.* 11:1283. 10.3389/fmicb.202001283 32625185 PMC7315799

[B37] Sambrook J. Russell D. W. (2001). *Molecular Cloning: A Laboratory Manual, Cold Spring Harbor Laboratory*, 3d Edn. New York NY: Cold Spring Harbor.

[B38] Silva L. Mourão J. Grosso F. Peixe L. (2018). Uncommon carbapenemase-encoding plasmids in the clinically emergent *Acinetobacter pittii*. *J. Antimicrob. Chemother.* 73 52–56. 10.1093/jac/dkx364 29069366

[B39] Smillie C. Garcillán-Barcia M. P. Francia M. V. Rocha E. P. de la Cruz F. (2010). Mobility of plasmids. *Microbiol. Mol. Biol. Rev.* 74 434–452. 10.1128/MMBR.00020-10 20805406 PMC2937521

[B40] Stokes H. W. Gillings M. R. (2011). Gene flow, mobile genetic elements and the recruitment of antibiotic resistance genes into Gram-negative pathogens. *FEMS Microbiol. Rev.* 35 790–819. 10.1111/j.1574-6976.2011.00273.x 21517914

[B41] Sullivan M. J. Petty N. K. Beatson S. A. (2011). Easyfig: a genome comparison visualizer. *Bioinformatics* 27 1009–1010. 10.1093/bioinformatics/btr039 21278367 PMC3065679

[B42] Thomas C. M. (2000). Paradigms of plasmid organization. *Mol. Microbiol.* 37 485–491. 10.1046/j.1365-2958.2000.02006.x 10931342

[B43] Touchon M. Cury J. Yoon E. J. Krizova L. Cerqueira G. C. Murphy C. (2014). The genomic diversification of the whole *Acinetobacter* genus: origins, mechanisms, and consequences. *Genome Biol Evol.* 6 2866–2882. 10.1093/gbe/evu225 25313016 PMC4224351

[B44] Turton J. F. Shah J. Ozongwu C. Pike R. (2010). Incidence of *Acinetobacter* species other than A. *baumannii* among clinical isolates of *Acinetobacter*: evidence for emerging species. *J. Clin. Microbiol.* 48 1445–1449. 10.1128/JCM.02467-09 20181894 PMC2849580

[B45] Veress A. Nagy T. Wilk T. Kömüves J. Olasz F. Kiss J. (2020). Abundance of mobile genetic elements in an *Acinetobacter lwoffii* strain isolated from Transylvanian honey sample. *Sci. Rep.* 10:2969. 10.1038/s41598-020-59938-9 32076091 PMC7031236

[B46] von Wintersdorff C. J. Penders J. van Niekerk J. M. Mills N. D. Majumder S. van Alphen L. B. (2016). Dissemination of antimicrobial resistance in microbial ecosystems through horizontal gene transfer. *Front. Microbiol.* 7:173. 10.3389/fmicb.2016.00173 26925045 PMC4759269

[B47] Wibberg D. Salto I. P. Eikmeyer F. G. Maus I. Winkler A. Nordmann P. (2018). Complete genome sequencing of *Acinetobacter baumannii* strain K50 discloses the large conjugative plasmid pK50a encoding carbapenemase OXA-23 and extended-spectrum β-lactamase GES-11. *Antimicrob. Agents Chemother.* 62 e212–e218. 10.1128/AAC.00212-18 29463529 PMC5923157

[B48] Zong Z. (2014). The complex genetic context of blaPER-1 flanked by miniature inverted-repeat transposable elements in *Acinetobacter johnsonii*. *PLoS One.* 9:e90046. 10.1371/journal.pone.0090046 24587208 PMC3934969

