# Supplementary material for: Ubiquitous Conjugative Mega-Plasmids of Acinetobacter Species and Their Role in Horizontal Transfer of Multi-Drug Resistance
Source: Front Microbiol. 2021 Sep 21;12:728644. doi: 10.3389/fmicb.2021.728644 (PMC8490738; doi:10.3389/fmicb.2021.728644)
Supplement: Supplementary Table 1 — Screening of pALWED1.1 plasmid among environmental Acinetobacter strains. [file Table_1.doc]

**Table S1.** Screening of pALWED1.1 plasmid among environmental *Acinetobacter* strains

| Strain | Resistance | Source | Hybridization with a probe for the *trbC* | PCR | |
| --- | --- | --- | --- | --- | --- |
|  |  |  |  | *trbC* | *rep* |
| ***A.lwoffii ** ED23-35** | Hg | Permafrost, river Homus-Yuryiah (20-40K years) | **+** | + | + |
| *A. johnsonii* ED45-25 | Hg | Permafrost, river Homus-Yuryiah (20-40K years) | **+** | + | + |
| *A. lwoffii* ED45-23 | Hg | Permafrost, river Homus-Yuryiah (20-40K years) | **-** | N/D*** | N/D |
| *Acinetobacter* sp. ED45-24 | Hg | Permafrost, river Homus-Yuryiah (20-40K years) | **-** | N/D | N/D |
| *Acinetobacter baumannii/ calcoaceticus* MR5-1 | Cm, Sm | Permafrost, Laptev sea (15-40K years) | **-** | N/D | N/D |
| *Acinetobacter baumannii/ calcoaceticus* MR5-8 | Sm | Permafrost, Laptev sea (15-40K years) | **-** | N/D | N/D |
| *Acinetobacter baumannii/ calcoaceticus* MR5-11 | Sm | Permafrost, Laptev sea (15-40K years) | **-** | N/D | N/D |
| *A. lwoffii* EK67 | Sm Sp Ap | Permafrost, river Homus-Yuryiah (20-40K years) | + | + | + |
| *A. johnsonii* M2-7 | Hg, Sm | Permafrost, river Homus-Yuryiah (1,9 mln years) | **-** | N/D | N/D |
| *Acinetobacter* sp. ED23 | Not found | Permafrost, river Homus-Yuryiah (20-40K years) | - | N/D | N/D |
| *A. lwoffii* ED28 | Not found | Permafrost, river Homus-Yuryiah (20-40K years) | + | + | + |
| *Acinetobacter* sp. ED37 | Not found | Permafrost, river Homus-Yuryiah (20-40K years) | - | N/D | N/D |
| *Acinetobacter* sp. ED72 | Not found | Permafrost, river Homus-Yuryiah (20-40K years) | - | N/D | N/D |
| *Acinetobacter* sp. M2-7 | Hg, Sm | Permafrost, river Homus-Yuryiah (20-40K years) | + | + | + |
| *A. baumannii/ calcoaceticus* Z3-16 | Hg | Kyrgyzstan (mercury mine) | + | + | + |
| *A. baumannii/ calcoaceticus* Z9-16 | Km | Kyrgyzstan (mercury mine) | +-** | - | N/D |
| *Acinetobacter* sp. A21 | Hg | Kyrgyzstan (mercury mine) | + | + | + |
| *Acinetobacter* sp. A116 | Hg | Kyrgyzstan (mercury mine) | - | N/D | N/D |
| *A. lwoffii* ARK5-14 | Sm, Sp | The Arctic, Vize Island, ground from the bottom of the lake | + | - | N/D |
| *Acinetobacter* sp. LS1-1 | Hg | Moscow region, soil | - | N/D | N/D |
| *Acinetobacter* sp. LS8-1 | Hg | Moscow region, soil | - | N/D | N/D |
| *Acinetobacter* sp. LS8-6 | Hg | Moscow region, soil | - | N/D | N/D |
| *Acinetobacter* sp. LS10-20 | Hg | Moscow region, soil | - | N/D | N/D |
| *Acinetobacter* sp. LS12-1 | Not found | Moscow region, water | + | + | + |
| *Acinetobacter* sp. LS12-25 | Hg | Moscow region, water | - | N/D | N/D |
| *Acinetobacter* sp. LS12-30 | Hg | Moscow region, water | - | N/D | N/D |
| *Acinetobacter* sp. LS18-1 | Hg | Moscow region, water | - | N/D | N/D |
| *Acinetobacter* sp. LS18-2 | Hg | Moscow region, water | - | N/D | N/D |
| *A. johnsonii* LS22-1 | Hg | Moscow region, water | - | N/D | N/D |
| *Acinetobacter* sp. LS22-3 | Hg | Moscow region, water | - | N/D | N/D |
| *Acinetobacter* sp. LS26-2 | Hg | Moscow region, water | - | N/D | N/D |
| *Acinetobacter* sp. LS33-1 | Hg | Moscow region, water | - | N/D | N/D |
| *Acinetobacter* sp. LS35-1 | Hg | Moscow region, water | - | N/D | N/D |
| *Acinetobacter* sp. LS45-3 | Hg | Moscow region, soil | + | - | N/D |
| *Acinetobacter* sp. LS46-1 | Hg | Moscow region, soil | + | N/D | N/D |
| *A. johnsonii* LS47-1 | Hg, Sm, Sp | Saratov, River Volga | - | - | - |
| *Acinetobacter* sp. LS55-9 | Hg | Moscow region, soil | + | + | + |
| *Acinetobacter* sp. LS56-7 | Hg | Moscow region, soil | + | + | + |
| *Acinetobacter* sp. LS57-6 | Hg | Moscow region, soil | + | - | N/D |
| *Acinetobacter* sp. LS57-7 | Hg | Moscow region, soil | - | N/D | N/D |
| *Acinetobacter* sp. LS3-27 | Hg | Moscow region, water | - | N/D | N/D |
| *Acinetobacter* sp. LS50-2 | Hg | Moscow region, water | - | N/D | N/D |
| *Acinetobacter* sp. LS53-7 | Hg | Moscow region, water | - | N/D | N/D |
| *Acinetobacter* sp. A120 | Hg | Kyrgyzstan (mercury mine) | + | - | N/D |
| *A. lwoffii* ANS7-7 | Sm, Sp, Ap, Tc | Antarctic, soil | +-** | + | + |
| *Acinetobacter* sp. MK63-5 | Hg | Kuril islands, Yuzhno-Kurilsk | + | - | N/D |
| *Acinetobacter* sp. TC1 | Hg | Carpathians, mercury mine | - | N/D | N/D |
| *A. lwoffii* TC41-3 | Hg | Carpathians, mercury mine | + | - | N/D |
| *Acinetobacter* sp. TC29-4 | Hg | Carpathians, mercury mine | - | N/D | N/D |
| *A. johnsonii* TC105 | Hg | Carpathians, mercury mine | + | + | + |
| *A. schindlerii* NC7-1 | Hg | Caucasus, mercury mine | + | - | N/D |
| *A. schindlerii* NC11-1 | Hg | Caucasus, mercury mine | + | - | N/D |
| *A. johnsonii* NC13-1 | Hg | Caucasus, mercury mine | + | + | + |
| *A. lwoffii* FA18-2 | Hg | Kamchatka Peninsula | + | - | N/D |
| *A. albensis* KHP18 | Hg | Kyrgyzstan (mercury mine) | + | + | + |
| *A. albensis* W14 | Hg | Kyrgyzstan (mercury mine) | +-** | + | + |
| *A. lwoffii* EL10-6 | Hg | Kamchatka Peninsula | - | N/D | N/D |

* The host strain of pALWED1.1; **+- - weak hybridization; *** ND – not determined

Antibiotics designations: Hg - HgCl_2_, Km - kanamycin, Ap - ampicillin, Sm - streptomycin, Sp - spectinomycin, Tc - tetracycline
