# Supplementary material for: Ubiquitous Conjugative Mega-Plasmids of Acinetobacter Species and Their Role in Horizontal Transfer of Multi-Drug Resistance
Source: Front Microbiol. 2021 Sep 21;12:728644. doi: 10.3389/fmicb.2021.728644 (PMC8490738; doi:10.3389/fmicb.2021.728644)
Supplement: Supplementary Table 2 — Identification of backbone region genes in genomes of modern plasmids related to pALWED1.1 (summary information for 27 modern plasmids). [file Table_2.doc]

**Table S2.** Identification of backbone region genes in genomes of modern plasmids related to pALWED1.1 (Summary information for 27 modern plasmids)

| Gene of pALWED1.1 | Coordinates in pALWED1.1 (bp) | Function of protein encoded by the corresponding gene | Identity level (%) |
| --- | --- | --- | --- |
| Putative *rep* | 228158-229948 | Replication initiator protein | 99,3-99,4 |
| *traY* | 273149-276157 | Integral membrane protein | 97,9-99,6 |
| *parA* | 296-1132 | Partitioning protein | 98,4-100 |
| *traJ* | 4484-5779 | Nucleotide binding protein | 99,0-100 |
| *traO* | 11372-12895 | Thick pilus formation | 98,2-99,9 |
| *traU* | 17222-20404 | Nucleotide binding protein | 98,9-99,9 |
| *trbC* | 20527-23319 | Coupling protein | 99,1-99,9 |
