# Supplementary material for: Ubiquitous Conjugative Mega-Plasmids of Acinetobacter Species and Their Role in Horizontal Transfer of Multi-Drug Resistance
Source: Front Microbiol. 2021 Sep 21;12:728644. doi: 10.3389/fmicb.2021.728644 (PMC8490738; doi:10.3389/fmicb.2021.728644)
Supplement: Supplementary Table 3 — Characteristics of Acinetobacter conjugative mega-plasmids. [file Table_3.doc]

**Table S3 Characteristics of *Acinetobacter*** conjugative mega-plasmids

| Strain | Plasmid (size) | Source | Country/Region | Mer-operons : mer2 mer 1 | Integron type | Resistance determinants to: | Accession no |
| --- | --- | --- | --- | --- | --- | --- | --- |
| *A.lwoffii* ED23-35 (prototype) | pALWED1.1 (287631 bp) | Permafrost | Russia:Kolyma | + + | - | Hg, Tc | KX426227.1 |
| *A.haemolyticus* TJR01 | pAHTJR1 (306131 bp) | Homo sapiens (respiratory patient) | China: Tianjin | - - | Ia | Km, Cb, Ap, Cm, Fr, Erm, Sul, Rif, Tc | CP038010.1 |
| *A.pittii* 2014N21-145 | p2014N21-145-1 (323995 bp) | Homo sapiens | Taiwan | + + | III | Hg, Km, Gm, Cb, Cm, Fr, Erm, Sul | CP033569.1 |
| *A.pittii* C54 | pC54_001 (256887) | Homo sapiens | Australia: Sydney | - - | II | Km, Gm, Ap, Cb, Erm, Sul, Tm, Cm, Fr | CP042365.1 |
| *A.johnsoni* Acsw19 | pAcsw19-2 ~ (351885) | Sewage | China: Luzhou | - + | - | Hg, Km, Ap, Cm, Erm | [CP043309.1](https://www.ncbi.nlm.nih.gov/nucleotide/CP043309.1?report=genbank&log$=nucltop&blast_rank=6&RID=UJWP584Y014) |
| *Acinetobacter* sp. WCHA55 | pOXA58_010055 (372328 bp) | Sewage | China: Sichuan, Chengdu | - + | Ia | Hg, Km, Gm, Cb, Ap, Cm, Erm, Sul, Rif | CP032285.1 |
| *A.baumannii* 34AB | p34AB (277864 bp) | Pig (caecum at slaughter) | China: Jiangsu | - - | - | Km, Gm, Erm, Cm, Fr, Tc | MK134375.1 |
| *A.pittii* 2014S07-126 | p2014S07-126-1 (284051 bp) | Homo sapiens | Taiwan | - - | I | Km, Gm, Cb, Ap, Sm, Sp, Erm, Sul, Rif | [CP033531.1](https://www.ncbi.nlm.nih.gov/nucleotide/CP033531.1?report=genbank&log$=nucltop&blast_rank=9&RID=UJWP584Y014) |
| *A.wuhouensis* WCHAW010062 | pOXA23_010062 (311749 bp) | sewage | China: Sichuan, Chengdu | - - | - | Km, Gm, Ap, Cb, Cm, Erm | [CP033130.1](https://www.ncbi.nlm.nih.gov/nucleotide/CP033130.1?report=genbank&log$=nucltop&blast_rank=10&RID=UJWP584Y014) |
| *A.defluvii* WCHA30 | pOXA58_010030 (355075 bp) | sewage | China: Chengdu, Sichuan | + + | I | Hg, Km, Gm, Ap, Cm, Erm, Sul, Rif | CP029396.2 |
| *A.johnsoni* XBB1 | pXBB1-9 (398857 bp) | Hospital sewage | China: Chengdu, Sichuan | + + | Ia | Hg, Km, Gm, Cb, Ap, Cm, Erm, Sul, Rif | CP010351.1 |
| *A.ursingii* RIVM0051 | pRIVM0051_IMP-4 (259278 bp) | Homo sapiens | Netherlands: Bilthoven | - - | II, V | Km, Gm, Ap, Erm,Sul, Tm, Cm | MH220286 |
| *A.ursingii* RIVM0002 | pRIVM0002_IMP-4 (317191 bp) | Homo sapiens | Netherlands: Bilthoven | - - | I, II,V | Km,Gm, Ap, Erm, Sul, Tm, Cm, Rif | MH220285 |
| *A.ursingii* RIVM0061 | pRIVM0061_IMP-4 (313407 bp) | Homo sapiens | Netherlands: Bilthoven | - - | I, II, V | Km, Gm, Ap, Erm, Sul, Tm, Cm, Rif | MH220287 |
| *A.pittii* AP43 | pAP43-OXA58-NDM1 (268263 bp) | Homo sapiens (urine) | China: Hangzhou | - - | - | Km, Cb, Gm, Ap, Erm, Cm, Fr | CP043053.1 |
| *A..baumannii* ABF9692 | pABF9692 (264805 bp) | trachea of Duck | China | - - | - | Cb,Cm, Erm, Sul, Tc, | CP048828.1 |
| *A..baumannii* E47 | pE47 (327867 bp) | Room 7 | Australia, Sydney | - - | II, V | Ap, Cb, Gm, Cm, Erm, Sul, Tm | CP042557.1 |
| *A..seiferti* AS4 | pAS4-1 (276086 bp) | Homo sapiens | Taiwan | - - | VI | Km, Cm, Cb, Ap, Gm Sul, Erm, Sm, Sp | CP061688.1 |
| *A..seiferti* AS23 | pAS23-1 (290682 bp) | Homo sapiens | Taiwan | - - | IV, VII | Km, Cm, Cb, Ap, Gm Sul, Erm, Tp, Fr, Sm, Sp | CP061673.1 |
| *A..seiferti* AS70 | pAS70-1 (281459 bp) | Homo sapiens | Taiwan | - - | IV | Km, Cb, Ap, Erm, Sul, Gm, Cm, Fr, Sm, Sp | CP061572.1 |
| *A..seiferti* AS74 | pAS74-1 (336046 bp) | Homo sapiens | Taiwan | + + | - | Hg, Km, Cb, Ap, Erm, Sul, Cm, Fr, Tc | CP061557.1 |
| *A.nosocomialis* WM98B | pWM08B (255232 bp) | Homo sapiens | Australia | + - | - | Hg, Km,Gm, Cm, Fr, Bc | MT742183 |
| *A.lwoffii* | pR4WN_12CE1 (270906 bp) | Prawn | East Australian Fisheries | - - | VIII | Gm, Tc, Sul, Sm, Sp | MT742180 |
| *Acinetobacter sp.TTH0-4* | pR4WN_1BD1 (284751bp) | Prawn | East Australian Fisheries | - - | VIII | Km, Gm, Sul, Sm, Sp | MT742182 |
| *A.johnsonii* | pR4WN_E10B (259080 bp) | Prawn | East Australian Fisheries | - - | VIII | Gm, Sul, Sm, Sp | MT742181 |
| *A.pittii* JXA13 | pHNJXA13-1 (206931 bp) | dog | China: Nanchang | - - | _ | Km, Cb, Gm, Erm, Tc, Sul | CP054138.1 |
| *A.baumannii* ABF9692 | pABF9692 (264805 bp) | trachea of Duck | China | - - | _ | Km, Cb, Cm, Tc, Sul | CP048828.1 |
| *A.*sp. CS-2 | unnamed2 (283930 bp) | hospital wastewater | China | - - | _ | Cb, Cm, Fr, Sul | CP067021.1 |

Hg – HgCl_2_; Tc - tetracyclin; Km – kanamycin; Gm – gentamycin; Cb – carbenicillin; Ap –ampicillin; Cm – chloramphenicol; Fr – florphenicol; Erm – erythromycin; Sul – sulphanilamide; Rif – rifampicin; Tm – trimethoprim; Sm – streptomycin; Sp – spectimomycin; Bc – bicyclomin
