# Supplementary material for: Ubiquitous Conjugative Mega-Plasmids of Acinetobacter Species and Their Role in Horizontal Transfer of Multi-Drug Resistance
Source: Front Microbiol. 2021 Sep 21;12:728644. doi: 10.3389/fmicb.2021.728644 (PMC8490738; doi:10.3389/fmicb.2021.728644)
Supplement: Supplementary Table 4 — rep and trbC genes of pALWED1.1 in whole genome shotgun sequences. [file Table_4.doc]

**Table S4.** *rep* and *trbC* genes of pALWED1.1 in whole genome shotgun sequences

| Strain | Source | Rep containing contigs (identity with *rep* of pALWED1.1, %), AC | *trbC* containing contigs (identity with *trbC* of pALWED1.1, %), AC |
| --- | --- | --- | --- |
| *A. lwoffii* AL_065 | bedside rail in hospital intensive care unit, Pakistan | NODE_4_length_109024_cov_29.2974, (99%), SEIL01000004.1 | NODE_4_length_109024_cov_29.2974, (99%), SEIL01000004.1 |
| *A. johnsonii* AJ_385 | washroom sink in hospital intensive care unit, Pakistan | NODE_17_length_66978_cov_40.762977, (100%), RHXE01000017.1 | NODE_21_length_51474_cov_41.171683, (99%), RHXE01000021.1 |
| *A. johnsonii* WCHAJo010049 17 | Sewage, China: Chengdu, Sichuan | -, (99%), SGVZ01000088.1 | -, (99%), SGVZ01000144.1, |
| *A. johnsonii* AJ_082 | washroom sink in hospital intensive care unit, Pakistan | NODE_8_length_87098_cov_29.8616, (99%), RHXI01000008.1 | NODE_28_length_44049_cov_30.2022, (99%), RHXI01000028.1 |
| *A. johnsonii* Aj2199 | peritoneal fluid, Homo sapiens, Argentina: Buenos Aires | contig_11, (99%), LVIB01000011.1 | contig_44, (99%), LVIB01000044.1 |
| *A. johnsonii* isolate S2_018_000_R3_109 | hospital room, USA | S2_018_000_R3_scaffold_24699, (100%), QFNG01000552.1 | S2_018_000_R3_scaffold_7733, (99%), QFNG01000249.1 |
| *A. pittii* TUM15415 | drain waste liquid, Japan: Tochigi | sequence022, (100%), BKSY01000022.1 | sequence037, (99%), BKSY01000037.1 |
| *A. pittii* NBRC 110510 | Hyogo College of Medicine Hospital, Japan | Alw02S_CON00048, BBTS01000048.1, (99%) | Alw02S_CON00039, (99%), BBTS01000039.1 |
| *A. pittii* UKK-0551 | Homo sapiens, Germany: somewhere in SN | SRR3547542.ctg000032, (99%), MDIP01000032.1 | SRR3547542.ctg000061, (99%), MDIP01000061.1 |
| *A. pittii* UKK-0550 | Germany: somewhere in TH, Homo sapiens | SRR3547541.ctg000176, (99%), MDIO01000176.1 | SRR3547541.ctg000155, (99%) MDIO01000155.1 |
| *A. pittii* UKK-0549 | Germany: somewhere in SN, Homo sapiens | SRR3547540.ctg000057, (99%), MDIN01000057.1 | SRR3547540.ctg000078, (99%), MDIN01000078.1 |
| *A. pittii* T167 | Homo sapiens, Thailand: Bangkok | T167_contig_49, (99%), JRQZ01000049.1 | T167_contig_78, (99%), JRQZ01000078.1 |
| *A. pittii* CR12-42 | Homo sapiens, Australia: Queensland | CR12-42_contig_37, (99%), JQNT01000036.1 | CR12-42_contig_42, (99%), JQNT01000041.1 |
| *A. pittii* AP1 | Sputum, Homo sapiens, Thailand: Roied | scaffold15, (99%), JAEFCT010000015.1 | scaffold15, (99%), JAEFCT010000015.1 |
| *A. pittii* 42F plasmid p2APIBUN42F | Hospital, Colombia | contig p2APISeq_003, (99%), CBRO020000082.1 | contig p2APISeq_013, (99%), CBRO020000092.1 |
| *A. pittii* TUM15587 | Catheter, Japan: Tokyo | sequence136, (99%), BKZG01000136.1 | sequence062 (100%), BKZG01000062.1 |
| *A. pittii*, TUM15431 | human pharyngeal swab, Japan: Tochigi | sequence122, (99%), BKTN01000122.1 | sequence045, (99%), BKTN01000045.1 |
| *A. pittii* TUM15428 | human urine, Japan: Tochigi | sequence008, (99%), BKTK01000008.1 | sequence008, (99%), BKTK01000008.1 |
| *A. pittii* TUM15016 | human urine, Japan: Tokyo | sequence034, (99%), BKDZ01000034.1 | sequence059, (99%), BKDZ01000059.1 |
| *A. pittii* NBRC 110514 | Drain, human, Japan: Osaka | sequence33, (99%), BJLJ01000033.1 | sequence18, (99%), BJLJ01000018.1 |
| *A. pittii* YMC2013/3/R2081 | Sputum, Homo sapiens, South Korea: Seoul | IonXpress_011_rawlib.basecaller_c125, (99%), MKHO01000030.1 | IonXpress_011_rawlib.basecaller_c78, (99%), MKHO01000143.1 |
| *A. nosocomialis* AN2605 | University Hospital of Abidjan-Yopougon, Homo sapiens, France | AN20_S68_R1__paired__contig_9 (99%), NNSH01000009.1 | AN20_S68_R1__paired__contig_9 (99%), NNSH01000009.1 |
| *A. nosocomialis* 28F | Hospital, Colombia | contig p2ANISeq_019 (99%), CBSD020000128.1 | contig p2ANISeq_014 (99%), CBSD020000123.1, |
| *A. nosocomialis* AN1 2 | Homo sapiens, China | - (99%), JACLCR010000002.1, **287897 bp** | - (99%), JACLCR010000002.1, **287897 bp** |
| *A. nosocomialis* Ab122 | Homo sapiens, Australia | NODE_17_length_10706_cov_23.015879 (99%), UCQC02000070.1 | NODE_1_length_128680_cov_21.486408, (99%), UCQC02000071.1 |
| *A. junii* WCHAJ010047 49 | Sewage, China: Chengdu, Sichuan | - (100%), SGST01000049.1 | - (99%), SGST01000026.1 |
| *A. junii* AJ_068 | washroom sink in hospital intensive care unit, Pakistan | NODE_5_length_94530_cov_24.2727, (99%), RHXJ01000005.1 | NODE_5_length_94530_cov_24.2727, (99%), RHXJ01000005.1 |
| *A. junii* AJ_351 | washroom sink in hospital intensive care unit, Pakistan | NODE_5_length_111866_cov_76.998461, (99%), RHXG01000005.1 | NODE_5_length_111866_cov_76.998461, (99%), RHXG01000005.1 |
| *A. junii* TUM15376 | human blood, Japan: Tokyo | sequence009 (99%), BKRL01000009.1 | sequence038 (99%), BKRL01000038.1 |
| *A. junii* NBRC 110497 | Culture collection NBRC:110497, Human sputum, Tokyo, Japan | contig Aju01S_CON00049 99%, BBOS01000049.1 | contig Aju01S_CON00035 99%, BBOS01000035.1 |
| *A. radioresistens* A154 | Antarctica: Fildes Peninsula | NODE_10_length_108528_cov_11.463 (99%), PXJD01000010.1 | NODE_10_length_108528_cov_11.463 (99%), PXJD01000010.1 |
| *A. radioresistens* A145 | Antarctica: Fildes Peninsula | NODE_3_length_107660_cov_8.02585 (99%), PXJE01000003.1 | NODE_3_length_107660_cov_8.02585 (99%), PXJE01000003.1 |
| *A. ursingii* TUM15522 | human blood, Japan: Kanagawa | sequence018 (100%), BKWY01000018.1 | sequence054 (99%), BKWY01000054.1 |
| *A. ursingii* isolate UBA5830 | Wood, terrestrial metagenome, USA: New York City | UBA5830_contig_149, (99%), DJHC01000052.1 | UBA5830_contig_253 (99%), DJHC01000012 |
| *A. ursingii* TUM15523 | human blood, Japan: Kanagawa | sequence030, (99%), BKWZ01000030.1 | sequence018 (99%), BKWZ01000018.1 |
| *A. ursingii* TUM15519 | human blood, Japan: Kanagawa | sequence041 (99%), BKWV01000041.1 | sequence043 (99%), BKWV01000043.1 |
| *A. ursingii* TUM15518 | human blood, Japan: Kanagawa | sequence014 (99%), BKWU01000014.1 | sequence035 (99%), BKWU01000035.1 |
| *A. ursingii* TUM15408 | human blood, Japan: Tokyo | sequence025 (99%), BKSR01000025.1 | sequence041 (99%), BKSR01000041.1 |
| *A. ursingii* TUM15098 | human blood, Japan: Tokyo | sequence049 (99%), BKHC01000049.1 | sequence028 (99%), BKHC01000028.1 |
| *A. ursingii* ANC 3649 | Acinetobacter ursingii NIPH ANC_3649 | acLru-supercont1.5.C24 (99%), APQC01000024.1 | acLru-supercont1.5.C28 (99%), APQC01000028.1 |
| *A. ursingii* TUM15489 | human blood, Japan: Tochigi | sequence023 (99%), BKVR01000023.1 | sequence010 (99%), BKVR01000010.1 |
| *A. bereziniae* KCTC 23199 | culture_collection KCTC:23199, Korea | Abe01S_CON02510, BBLJ01000029.1 (99%) | contig: Abe01S_CON03910 (99%), BBLJ01000045.1 |
| *A. bereziniae* LMG 1003 = CIP 70.12 | type strain | acLsv-supercont1.1.C2 (99%) APQG01000002.1 | acLsv-supercont1.1.C9 (99%), APQG01000009.1 |
| *A. towneri* AeBJ009 | hospital sewage, China | NODE_68_length_10846_cov_17.445_ID_143068 (99%), SIST01000066.1 | NODE_1_length_109026_cov_13.9675_ID_142934 (99%), SIST01000001.1 |
| *A. sichuanensis* WCHAc060041 | Sewage, China: Chengdu, Sichuan | 47 (100%), PYIX02000047.1 | 14 (99%), PYIX02000014.1 |
| *A. baumannii* S11 | Clinical?, Singapore | S11_contig_12 (100%), LAIY01000011.1 | S11_contig_33 (99%), LAIY01000029.1 |
| *A. baumannii* MRSN22112 | Blood Homo sapiens, Peru | MRSN22112_contig00014 (99%), VHGJ01000014.1 | MRSN22112_contig00016 (99%), VHGJ01000016.1 |
| *A. baumannii* 4300STDY7045708 | Homo sapiens, Thailand | ERS1930182SCcontig000024 (99%), UFIO01000024.1 | ERS1930182SCcontig000023 (99%), UFIO01000023.1 |
| *A. baumannii* BAuABod-3 | Turkey, Germany | NODE_15_length_75946_cov_30.566 (99%), NIWN01000015.1 | NODE_67_length_20939_cov_42. 4091 (99%), NIWN01000067.1 |
| *A. baumannii* 269 | Mucoid Sputum, Homo sapiens, Malaysia | contig_74 (99%), JQNV01000074.1 | contig_48 (99%), JQNV01000048 |
| *A. baumannii* 573719 | Sputum, Homo sapiens, USA | ab573719.contig.8_1 (99%), JFYA01000009.1 | ab573719.contig.8_1 (100%), JFYA01000009.1 |
| *A. baumannii* 13ARS_CVM0021 | NCBI Pathogen Detection Project | SAMEA25870168-rid6941973.denovo.18 (99%), DADBIT010000017.1 | SAMEA25870168-rid6941973.denovo.18 (99%), DADBIT010000032.1 |
| *A. baumannii* NCGM 202 | Homo sapiens, Viet Nam: Ho Chi Minh | SAMD00030179-rid8877773.denovo.137 (99%), DADAXM010000134.1 | SAMD00030179-rid8877773.denovo.046 (99%), DADAXM010000046.1 |
| *A. baumannii* A134 SAMN13028174- | Sputum, Homo sapiens, China: Wulumuqi | rid9741963.denovo.035 (99%), DADAQB010000032.1 | rid9741963.denovo.045 (99%), DADAQB010000041 |
| *A. baumannii* TUM15094 | human blood, Japan: Tokyo | sequence008 (99%), BKGY01000008.1 | sequence008 (99%), BKGY01000008.1 |
| *A. baumannii* NBRC 110492 | Japan: Hyogo, Human urine | contig: Aba23S_CON00080 (99%), BBTD01000080.1 | Aba23S_CON00049, 99%, BBTD01000049 |
| *A. baumannii* Ab06 | Blood, Homo sapiens, Brazil: Hospital das Clinicas, Sao Paulo | contig00072 (99%), LMBM01000072.1 | contig00050 (99%), LMBM01000050.1 |
| A. baumannii CCBH28083 7 | Blood, Homo sapiens, Brazil | - (99%), JAECSJ010000007.1 | - (99%), JAECSJ010000026 |
| A. variabilis SCsl29 | Liver, pig, China | NODE_27_length_51061_cov_122.262 (99%), VDIJ01000027.1 | NODE_23_length_54881_cov_121.797 (99%), VDIJ01000023.1 |
